# The transcriptional regulation of ciliogenesis in differentiating Drosophila sensory neurons

**DOI:** 10.1186/2046-2530-1-S1-O14

**Published:** 2012-11-16

**Authors:** A Jarman, FG Newton, PI zur Lage, G Gallone, DJ Moore, K Styczynska

**Affiliations:** 1 University of Edinburgh, UK

## 

In contrast to the progress in understanding ciliogenesis and cilium function, we know less about the transcriptional regulation of ciliogenesis genes and how this regulatory program is modulated to generate diverse cilia. *Drosophila* sensory neurons have ciliary dendrites that are structurally and functionally specialised for receiving different sensory modalities. Time-course gene expression profiling of differentiating chordotonal (Ch) mechanosensory neurons allowed us to determine how Atonal, a proneural bHLH factor, regulates events leading to mechanosensory cilium formation and specialisation. Atonal regulates ciliogenesis via activation of two downstream transcription factors: the well-known cilia gene regulator, Rfx, and a novel factor of the Forkhead family (Fd3F). Rfx regulates a variety of ciliogenesis genes in all ciliated sensory neurons. In contrast, Fd3F is unique to Ch neurons, where it regulates a cohort of genes required for ciliary motility – a unique specialisation of Ch cilia in *Drosophila* and an essential part of the hearing mechanism. Among the targets of Fd3F are genes with human homologues linked to primary ciliary dyskinesia, a congenital condition resulting from defective ciliary motility. We provide evidence that Fd3F is a previously unrecognised orthologue of Foxj1, the so-called master regulator of motile ciliated cells in vertebrates. Interestingly, Fd3F and Rfx cooperate to regulate motility target genes directly via adjacent DNA binding sites, thus providing a mechanism for how Rfx can regulate genes for cilium specialisation in addition to ‘core’ ciliogenesis genes. Our study suggests how Foxj1 and Rfx factors may interact in vertebrates to generate ciliary diversity.

http://www.ed.ac.uk/schools-departments/integrative-physiology/staff-profiles/andrew-jarman

